# Prevalence of depressive symptoms in patients with advanced schistosomiasis in China: A systematic review and meta-analysis

**DOI:** 10.1371/journal.pntd.0012003

**Published:** 2024-03-07

**Authors:** Yu-Xin Qi, Meng-Rui Huang, Hui-Ying Sun, Xiao-Yan Wu, Ze-Ting Liu, Da-Bing Lu

**Affiliations:** Department of Epidemiology and Statistics, School of Public Health, Jiangsu Key Laboratory of Preventive and Translational Medicine for Geriatric Diseases, MOE Key Laboratory of Geriatric Diseases and Immunology, Suzhou Medical College of Soochow University, Suzhou, Jiangsu, People’s Republic of China; Federal University of Ceará, Fortaleza, Brazil, BRAZIL

## Abstract

**Background:**

Advanced schistosomiasis is the most serious outcome of infection and has a negative impact on both physical fitness and mental health of patients, the latter of which has long been overlooked. Therefore, we performed this systematic review and meta-analysis to estimate the overall prevalence of depressive symptoms, one of the most common mental problems, in patients with advanced schistosomiasis in China.

**Methods:**

Six electronic databases were searched for studies reporting the prevalence of depressive symptoms in the targeted patients. Assessments were pooled using a fixed- or random-effects model based on heterogeneity test. Subgroup analyses were further performed and differences between/among groups were examined using the chi-squared test. The protocol had previously been registered in PROSPERO (CRD42023406708).

**Results:**

A total of 11 studies with 1,673 participants were included. The pooled prevalence of depressive symptoms in advanced schistosomiasis in China was 62.01% (95% CI: 51.30% - 72.72%), with a significant heterogeneity among studies. Depressive symptoms were more prevalent in patients with complications and more than half of the patients suffered a mild- or moderate-level of depression. No publication bias was found, and sensitivity analysis showed a stable result.

**Conclusions:**

The overall prevalence of depressive symptoms in advanced schistosomiasis in China was high enough to warrant psychotherapeutic interventions, especially for patients with complications. This would greatly prevent or/and reduce depression and improve their quality of life.

## Author summary

Schistosomiasis is a neglected tropical disease (NTD) which affects more than 250 million people across 78 countries worldwide. The three major schistosomes infecting humans are *Schistosoma mansoni*, *S*. *japonicum*, and *S*. *haematobium*. In China, after 70 years of control efforts, tremendous progress has been made, and now the number of *schistosomiasis japonica* infections is at its lowest historical level. However, advanced schistosomiasis, which is the most serious outcome of schistosome infection, poses a great threat as an increasing number of the patients have been diagnosed. Depression is a frequently occurring mental health issue caused by the disease that has long been overlooked. Therefore, we performed this meta-analysis to estimate the overall prevalence of depressive symptoms in patients with advanced schistosomiasis in China and its possible influential factors. We found that the pooled prevalence was 62.01% (95% CI: 51.30% - 72.72%) in the targeted patients and the estimate was associated with complications of the disease. Our study would call for psychological interventions to improve quality of life for patients with advanced schistosomiasis.

## Introduction

Schistosomiasis is considered to be a neglected tropical disease (NTD) [1]. As the third most devastating tropical disease, it affects over 230 million people worldwide [2]. Of the three main schistosome species that can infect humans (i.e., *Schistosoma japonicum*, *S*. *mansoni* and *S*. *haematobium*), *S*. *japonicum* causes the most severe pathological lesions [3,4] due to its greatest egg output, which deposit around the portal vein, causing blood flow obstruction and leading to the development of portal hypertension, collateral vessels, and splenomegaly [5]. In addition to substantial enlargement of the spleen and liver as well as intestinal lesions, it also causes focal cerebral symptoms and encephalic disease [6–8]. China is an endemic area for *S*. *japonicum* and was once one of the four countries most affected by *S*. *japonicum* in the world [9]. A survey in 1949 estimated that 11.6 million people in China were infected with schistosomiasis [10]. Thanks to the control work accessed by the central government for the past seven decades, the number of schistosome infections has decreased significantly over time and the schistosomiasis epidemic in China is currently at its lowest historical level. Nowadays, among the 12 endemic provinces in China, five provinces have achieved the level of transmission interruption and seven have achieved the level of transmission control [11]. However, the incidence of advanced schistosomiasis has been steadily increasing due to the persistent progress of the disease when without proper or/and timely treatment. For example, in 2020, out of 19,214 patients treated for schistosomiasis, a total of 19,209 cases (up to 99.97%) were diagnosed with advanced schistosomiasis [12]. The increasing number of advanced schistosomiases has raised a significant challenge to achieving the goal of schistosomiasis elimination in China by 2030 [13].

Human schistosomiasis caused by *S*. *japonicum* is divided into three phases: acute phase, chronic phase, and advanced phase. Advanced schistosomiasis is the last stage and also the most serious outcome of infection. It can occur when acute or chronic schistosomiasis is not treated effectively and timely or people with long-term recurrent infections, as adult worms can survive for years (or for decades) in mesenteric veins encrusted with host antigens [1,14–16]. It has also been reported that advanced schistosomiasis can occur even after the patients had been cured for more than 20 years [17]. There are currently four major types of advanced schistosomiasis in China, with two types (i.e., ascites and megalosplenia) being the most frequently reported [18,19]. Most patients are associated with severe disability and poor quality of life due to reduced aerobic capacity and complications related to impaired liver function [20], causing a heavy disease burden [21–23].

Advanced schistosomiasis has emerged as a risk factor for psychological morbidity, specifically depression, highlighting the importance of addressing mental health concerns in conjunction with the treatment of physical ailments [24]. The occurrence of depressive symptoms in advanced schistosomiasis patients has been frequently reported and is often linked to concerns regarding the prognosis of the disease [18,25,26]. Fang *et al*. found that 83.05% of patients with advanced schistosomiasis had more positive items (i.e., depression, anxiety, terror and somatization) than normal based on the Self-Rating Anxiety Scale (SAS) and the Self-Rating Depression Scale (SDS) [27]. However, the overall prevalence of depressive symptoms in the target patients remains unclear. Therefore, in order to provide information on the prevalence of the depressive symptoms caused by *S*. *japonicum* infection in advanced schistosomiasis patients in China and its possible influencing factors, we performed this systematic review and meta-analysis to synthesize the results of all relevant studies.

## Methods

### Registration and reporting

The protocol of this study has been previously registered in PROSPERO (CRD42023406708) and this study also followed the Preferred Reporting Items for Systematic Reviews and Meta-Analyses (PRISMA) guidelines [28].

### Search strategy

We searched Chinese databases including China National Knowledge Infrastructure (CNKI), WanFang Data Knowledge Service Platform (WanFang Data), China Science and Technology Journal Database (CQVIP) and English databases including PubMed, The Cochrane Library and Web of Science for studies reporting data on the prevalence of depression in advanced schistosomiasis until November 5, 2023. The search strategy of the Chinese databases was "wanqixuexichongbing" AND ("yiyu" OR "xinli") and that of the English databases were "advanced schistosomiasis" and "depression". Meanwhile, the reference lists of all included studies were manually searched for relevant studies.

### Inclusion criteria

The review included all articles that met all of the following criteria:

- Studies conducted in mainland China.
- Patients with advanced schistosomiasis were diagnosed by schistosomiasis control units or specialist hospitals according to the Diagnostic Criteria for Schistosomiasis (WS261-2006) [29].
- Articles reported the depressive symptoms prevalence or provided data that allowed the estimate to be calculated.
- Depressive symptoms were diagnosed with a standard scale such as SDS, SCL-90, or EQ-5D-based series.

In the case of duplicate publications identified, only the latest studies among them or those with the most complete data were selected. Articles in Chinese or English were retrieved, and were excluded if they did not meet any of the above criteria. There was no restriction on year of publications.

### Data extraction

Literature extraction was performed by two independent reviewers (YQ and MH). The following information was extracted from each study by using a standardized form: the first author, publication year, research year, province where study was conducted, study design, mean age of participants (standard deviation, SD), sample size, diagnostic method for depressive symptoms, prevalence rate of depressive symptoms reported, degree of depression, as well as possible factors influencing depression prevalence (patients’ gender, marital status, and with complications or not). In case of disagreement, a third reviewer (HS) was referred to and consulted for consensus. The content extraction of the articles was recorded in Excel.

### Study quality assessment

Two independent reviewers critically assessed the quality of each included study using the Joanna Briggs Institute’s Critical Appraisal Checklist for Prevalence and Incidence Studies [30]. The checklist consisted of nine questions each with four options including yes and no. A ’Yes’ means that the standard was met and a score of 1 would be awarded. Otherwise, a score of 0 would be assigned. A maximum number of one point for every numbered item would have been awarded to each study. A paper with a total score of ≥ 7 points was considered to be of high quality. Any discrepancies in the quality scores were resolved by further discussion with other team members. EndNote 20 was used to organize the identified articles.

### Statistical analysis

Statistical analysis was performed using R version 4.2.2 (https://www.R-project.org/) and R studio (https://www.rstudio.com/) with the ’meta’ package [31]. The prevalence of depressive symptoms was estimated by pooling the primary data from the included articles. A random or fixed effects model was used to estimate the prevalence with its 95% confidence interval based on the heterogeneity of all included studies. Heterogeneity was assessed using the Cochran *Q* test (*p* < 0.1 indicated an existence of heterogeneity) and the *I*^*2*^ statistic. The latter takes values from 0 to 100%. It was assumed that *I*^*2*^ values of 25%, 50% and 75% represented for low, moderate and high heterogeneity, respectively [32]. However, as *I*^*2*^ statistic is not able to give a true picture of heterogeneity, we therefore computed prediction intervals to reflect the heterogeneity of this meta-analysis [33]. If the presence of significant heterogeneity was suggested, a subgroup analysis was done to explore the sources of heterogeneity. It would be conducted according to study period (2018–2022, 2013–2017 or 2004–2012), study design (cross-sectional or clinical trial), region (province), diagnostic method for depressive symptoms, depression level (mild, moderate or severe), gender (male or female), marital status (married or unmarried) and complications (yes or no). Before pooled analysis, the data of prevalence values were decided whether to be transformed with Freeman-Tukey double arcsine, arcsine, log, logit or not based on their distribution [34]. If not following the normal distribution, arcsine transformation can, when some values are either small or large, be applied. Double arcsine, known as Freeman-Tukey double arcsine, is well-suited for the originally intended purpose of providing a variance stabilizing transformation for a (single) proportion, which is superior to the arcsine transformation. However, this transformation has general issues of monotonicity and invertibility [35]. The log and logit transformations are frequently used with their formulas being more straightforward [36].

The publication bias was assessed by visual inspection of the asymmetry of the funnel plot, and its significance was examined with both Egger’s test [37] and Begg’s test [38] for certainty. Sensitivity analysis was carried out by excluding studies one at a time. Statistical tests were two-sided with a significance threshold of *p* < 0.05.

## Results

### Search results

**Fig 1:**
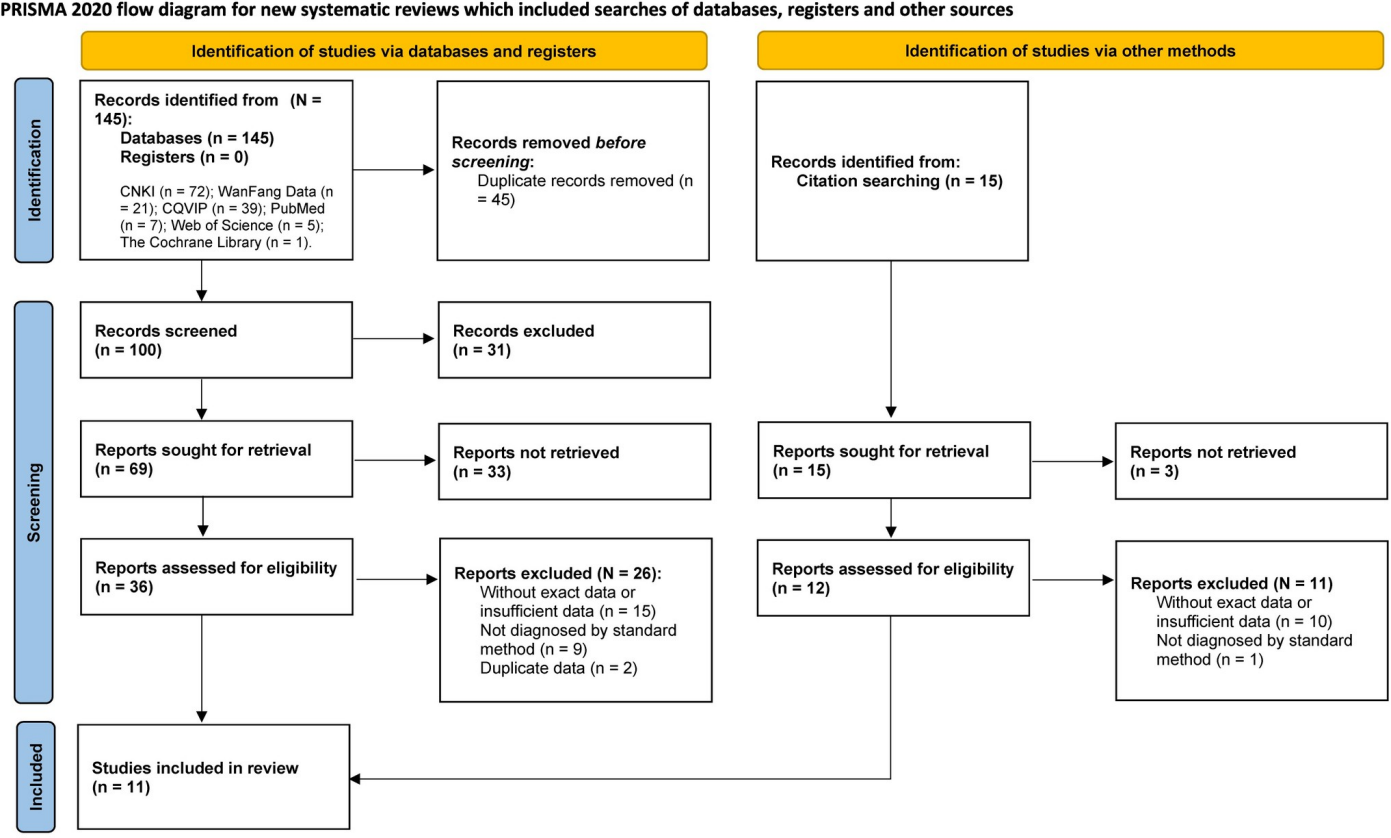
PRISMA flow diagram of search results and study selection [28].

The flow diagram is shown in Fig 1. A total of 160 relevant articles were obtained, of which 45 articles were duplicates and removed. After screening the titles and abstracts of the remaining 115 articles, 67 were further excluded. 48 full-text articles were then assessed for eligibility. Of these, 25 articles had insufficient data, ten were not assessed by a standard method for depression, and two had duplicate data. Finally, 11 articles [21,39–48] with a total of 1,673 participants were included in this work.

### Study characteristics and quality assessment

The characteristics of the 11 included studies are presented in Table 1. Nine were classified into cross-sectional design and two into clinical trial. The regions where the 11 studies were performed covered four provinces in China. Out of 1,673 participants with advanced schistosomiasis, 1,068 were diagnosed with depression problems. Five standard scales were used with SDS in six studies, SCL-90 in two studies and the other three (EQ-5D-5L, EQ-5D plus, and EQ-5D+C, all based on EQ-5D) each in only one of the remaining studies. The prevalence of depressive symptoms in advanced schistosomiasis ranged from 30.30% to 86.60% among studies. The quality of all included studies for this meta-analysis was rated as high because all scored ≥ 7 points according to the JBI checklists (see details in S2 Table).

**Table 1:** Selected characteristics of the 11 studies included in this systematic review and meta-analysis.

First author, year	Region	Year of study performed	Study design	Diagnostic method for depressive symptoms	Mean Age (SD)	Depressive symptoms prevalence % (depressions/participants)	JBI score
Wang, 2022 [39]	Jiangxi	March 2019—March 2022	Cross-sectional	SDS	54.36 (8.07)	41.67 (50/120)	7
Liu, 2021 [40]	Zhejiang	2020	Cross-sectional	EQ-5D-5L	75.04 (7.53)	62.40 (78/125)	7
Zhou, 2020 [41]	Hunan	December 2018—November 2019	Clinical trial	SDS	59.72 (11.63)	86.60 (84/97)	7
Pan, 2014 [42]	Hunan	January 2012—December 2013	Clinical trial	SDS	45.03 (9.74)	30.30 (20/66)	8
Zhou, 2014 [43]	Hunan	January 2012—December 2013	Cross-sectional	SDS	58.02 (12.51)	69.40 (143/206)	7
Jia, 2011 [21]	Hunan	October 2007—January 2008	Cross-sectional	EQ-5D plus	57.1 (12.6)	80.90 (174/215)	8
Nie, 2011 [44]	Jiangxi	April 2005—August 2010	Cross-sectional	SDS	NR	48.69 (149/306)	7
Deng, 2008 [45]	Hunan & Hubei	2007	Cross-sectional	EQ-5D+C	NR	76.07 (248/326)	8
Xiong, 2008 [46]	Jiangxi	2004	Cross-sectional	SCL-90	55.4 (NR)	58.33% (35/60)	7
Huang, 2006 [47]	Hubei	April 2004—March 2005	Cross-sectional	SDS	57 (NR)	47.06 (48/102)	7
Xiao, 1997 [48]	Hunan	March 1993—October 1993	Cross-sectional	SCL-90	49 (13)	78.00 (39/50)	7

Abbreviations: SDS, Self-Rating Depression Scale; EQ-5D-5L, EuroQol-5 Dimension-5 Level Questionnaire; EQ-5D plus, the EQ-5D extended with a cognitive dimension; EQ-5D+C, the EQ-5D extended with a cognitive dimension; SCL-90, the Symptom Check List 90; NR, not reported.

### Overall prevalence of depressive symptoms

**Fig 2:**
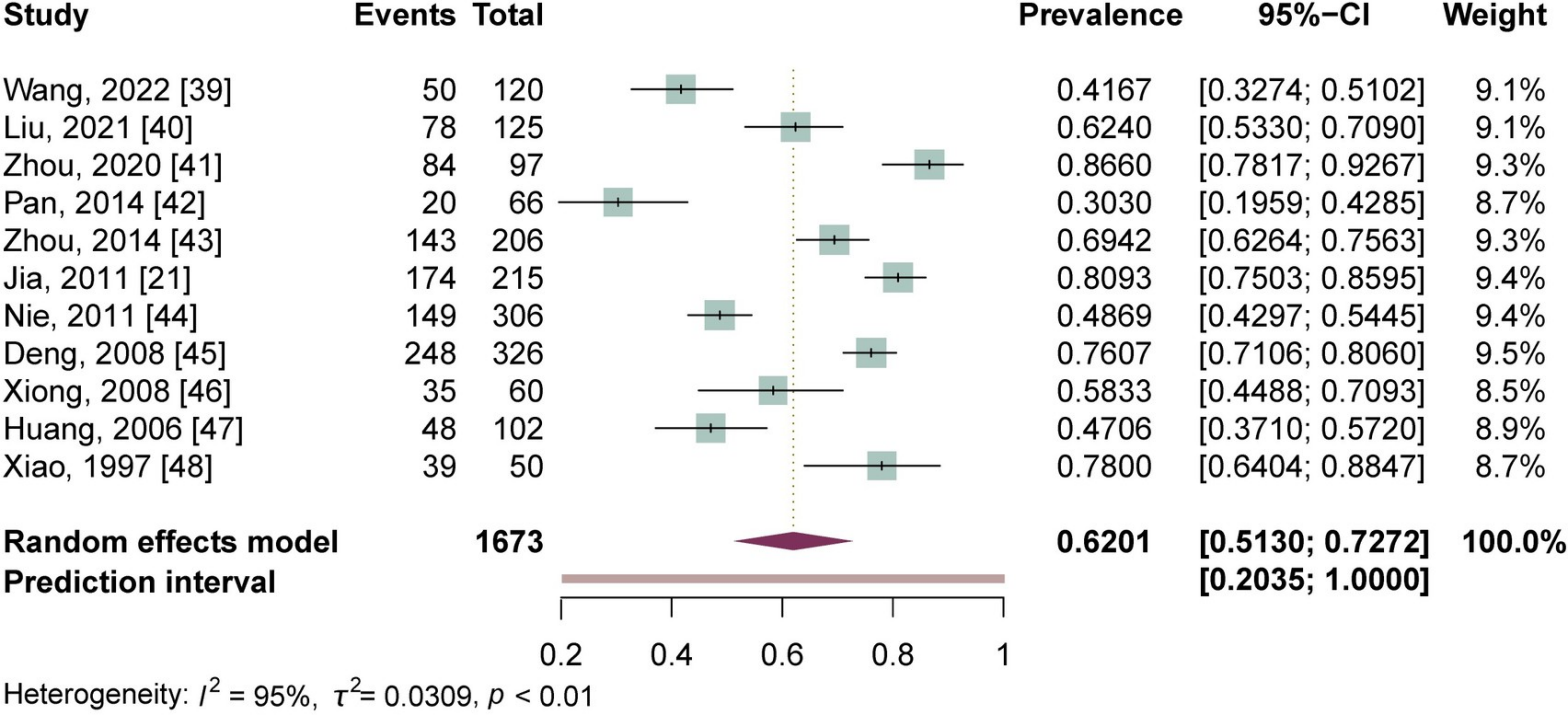
Forest plot of the prevalence of depressive symptoms in advanced schistosomiasis (n = 1,673). 95% CI, 95% confidence interval.

As all values of the depressive symptoms prevalence of individual studies were not close to 0 or 1, and also followed the normal distribution (W = 0.96, *p* = 0.73), no transformation for the prevalence data were needed in this meta-analysis. Due to the existence of the high heterogeneity among the included studies (*Q* = 212.02, *p* < 0.01; *I*^*2*^ = 95%, *p* < 0.01), a random effects model was used to estimate the overall prevalence of depressive symptoms in advanced schistosomiasis. The pooled estimate was 62.01% (95% CI: 51.30%-72.72%). The prediction interval was estimated to be 20.35% to 100%, which means that the true prevalence of depressive symptoms in 95% of all advanced patients would fall within this interval. The forest plot for the pooled prevalence of all included studies is shown in Fig 2.

### Subgroup analyses

**Fig 3:**
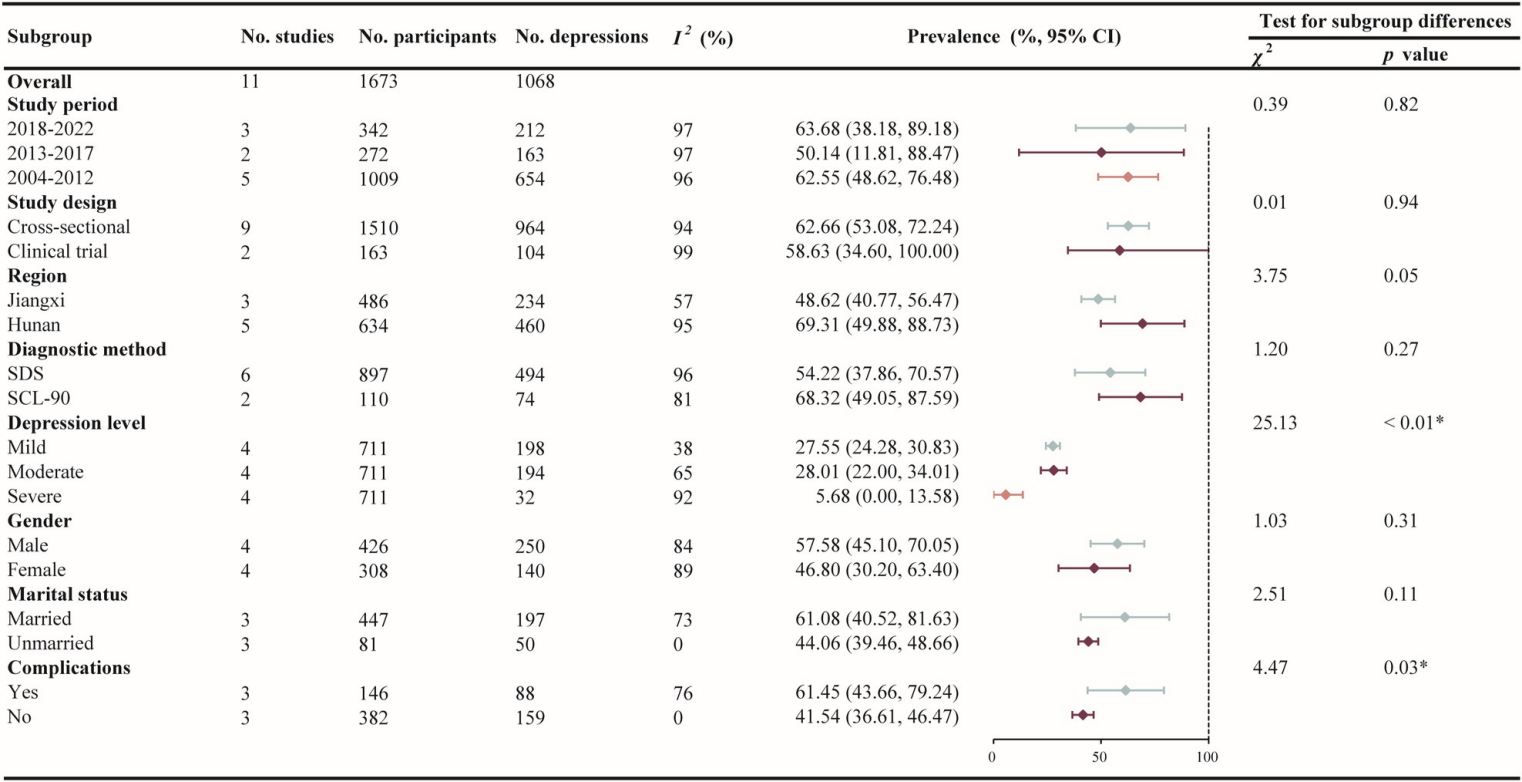
Subgroup analyses of prevalence (%, 95% CI) of depressive symptoms in advanced schistosomiasis. Four papers provided information on the level of depression, four on genders, three on marital status and three on complications of the disease. * *p* < 0.05.

**Fig 4:**
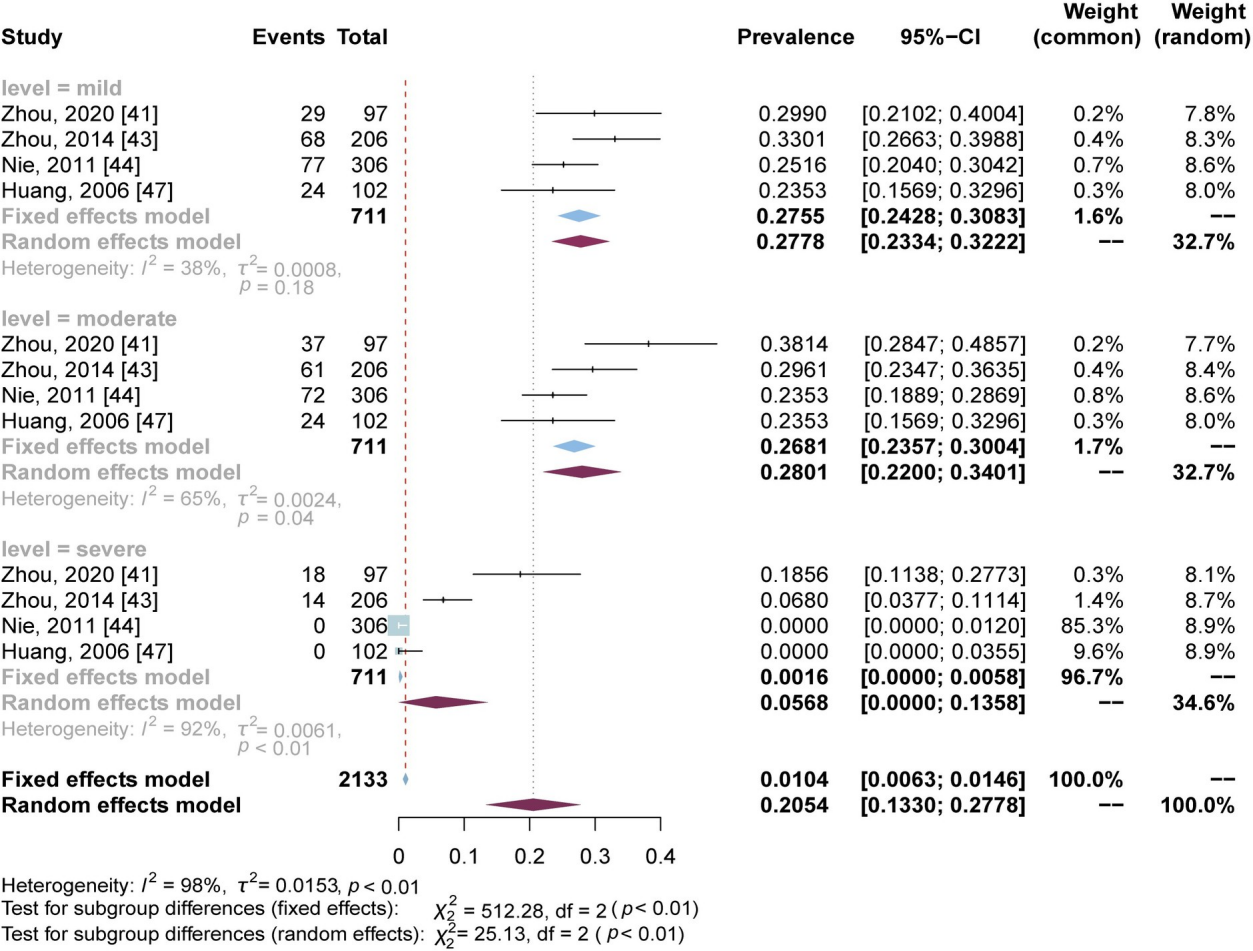
Forest plot of depressive symptoms prevalence stratified by the level of depression.

**Fig 5:**
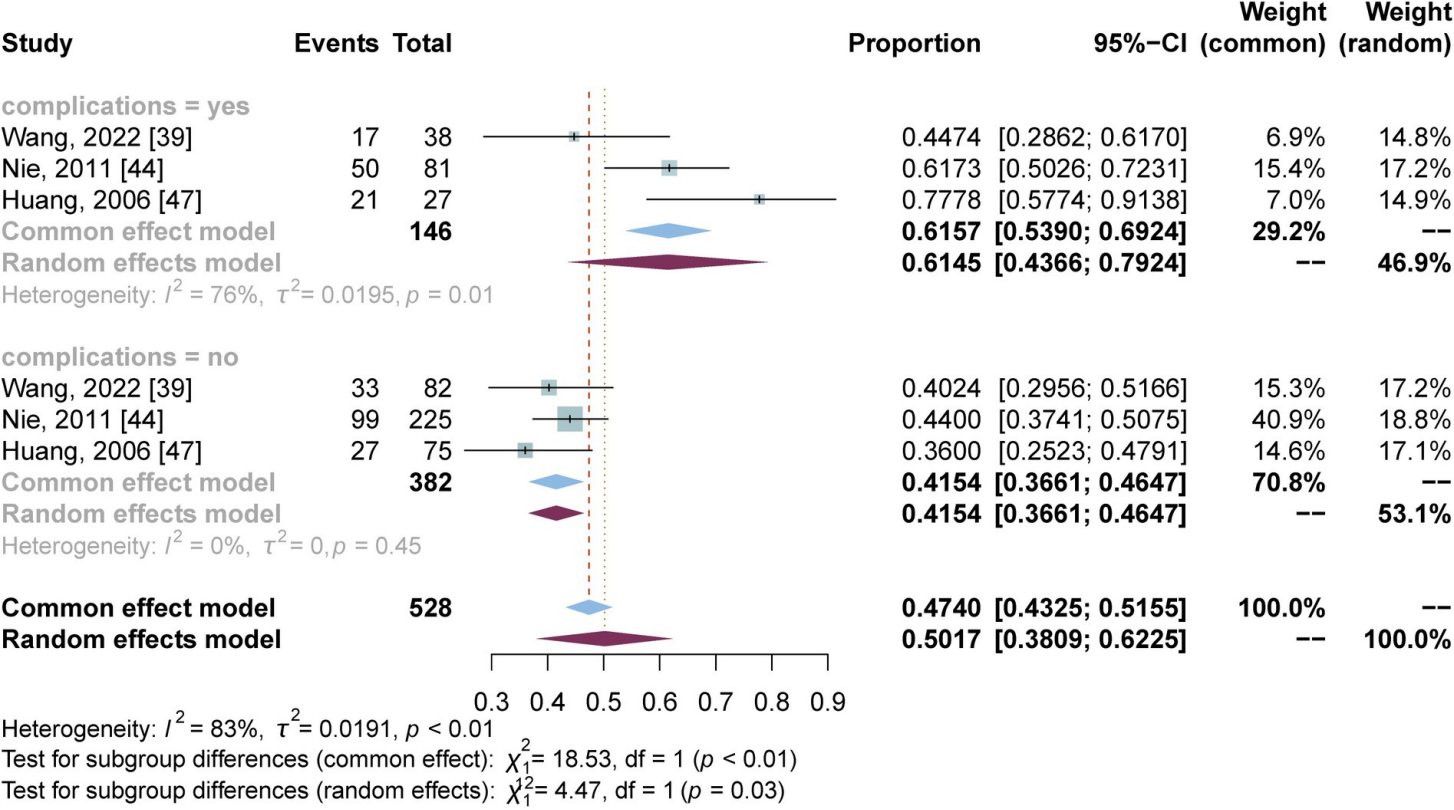
Forest plot of depressive symptoms prevalence stratified by suffering complications or not.

As seen in Fig 3, to explore the possible sources of heterogeneity, subgroup analyses were performed based on study period, study design, diagnostic method for depressive symptoms, region, depression level, gender, marital status and complications, respectively. Significant difference was observed only among/between subgroups when based on depression level and with complications or not. Four studies [41,43,44,47] provided prevalence data on the degree of depression (Fig 4). The subgroup meta-analyses showed that their prevalence and confidence intervals of three depression levels (mild, moderate or severe) were 27.55% (95% CI: 24.28% - 30.83%), 28.01% (95% CI: 22.00% - 34.01%) and 5.68% (95% CI: 0% - 13.58%), respectively, with *p* < 0.01 among them. Three studies provided data on participants with complications or not (Fig 5). Analysis showed that advanced schistosomiasis suffering from complications had a slightly higher depression prevalence (61.45%, 95% CI: 43.66%-79.24%) than those with no complications (41.54%, 95% CI: 36.61%-46.47%), with a significant difference (*p* = 0.03). The results above confirmed that depression level and with complications or not are moderators of depressive symptoms prevalence for advanced schistosomiasis patients.

### Publication bias and sensitivity analyses

**Fig 6:**
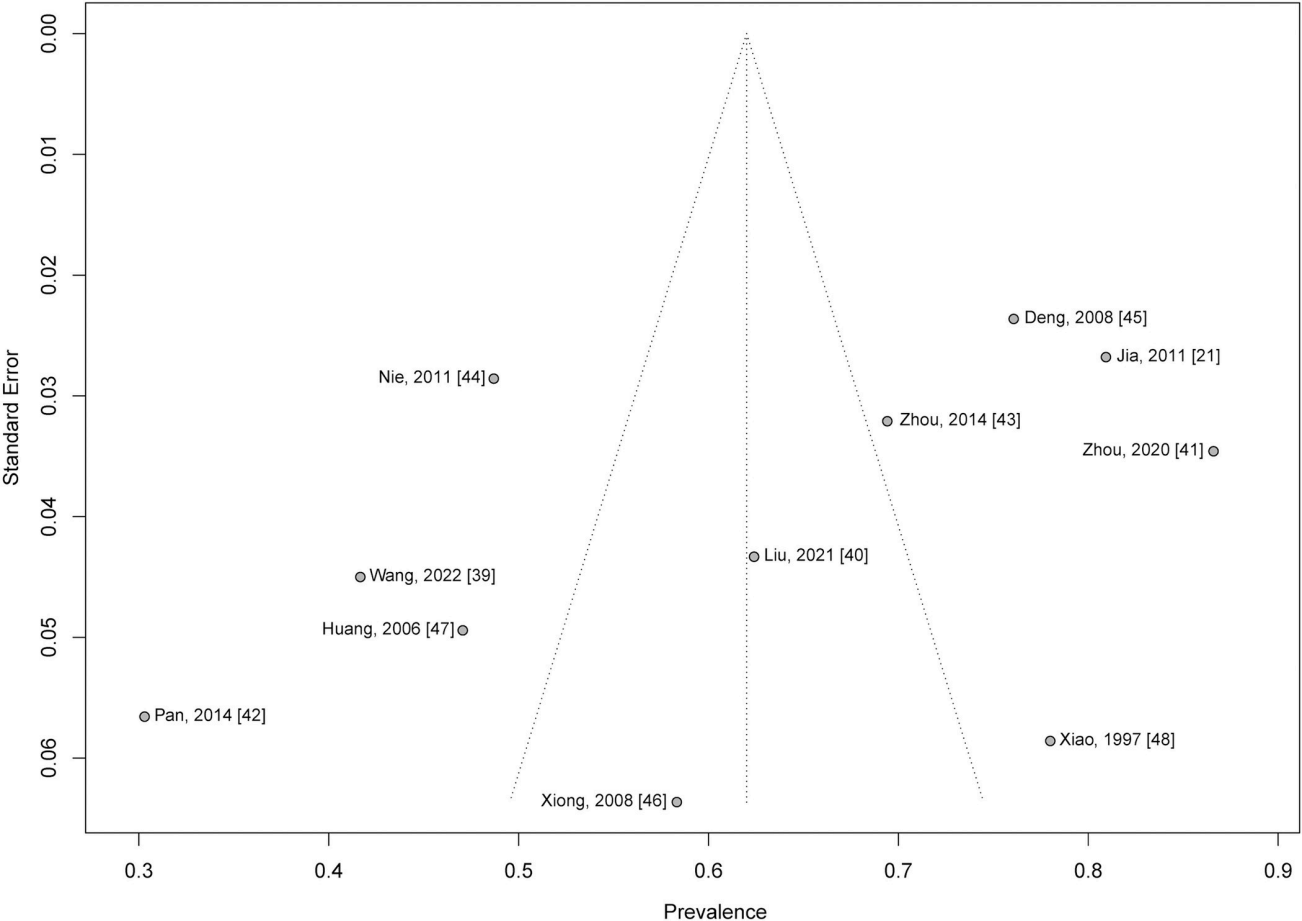
Funnel plots of the prevalence of depressive symptoms in advanced schistosomiasis.

**Fig 7:**
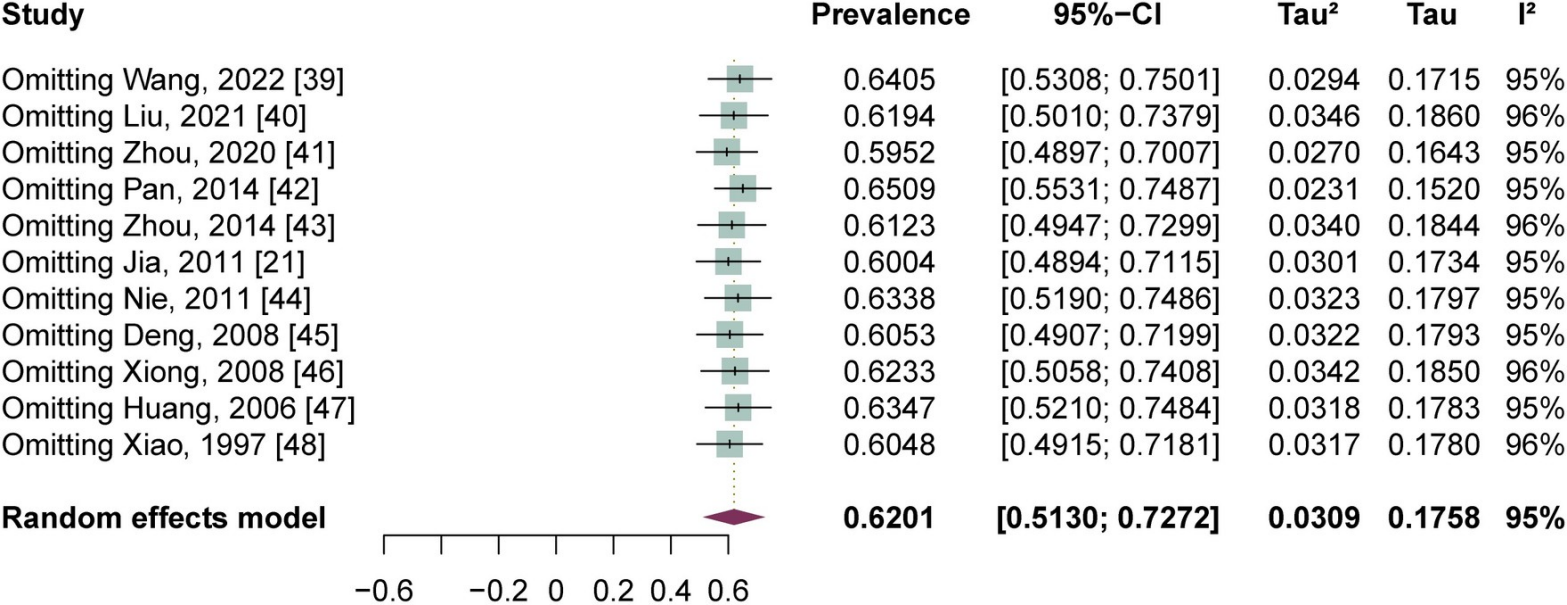
Sensitivity analysis of the prevalence of depressive symptoms in advanced schistosomiasis.

A funnel plot was conducted to evaluate publication bias (Fig 6). Both the Egger’s test (t = -1.62, *p* = 0.14) and the Begg’s test (z = -0.93, *p* = 0.35) showed no evidence of publication bias. Sensitivity analysis revealed that the pooled prevalence after excluding each study in turn varied between 59.52% (95% CI: 48.97% - 70.07%) and 65.09% (95% CI: 55.31% - 74.87%), which were similar to the above overall estimate, confirming robustness of the main analyses. See Fig 7.

## Discussion

In this meta-analysis, we aimed to estimate the overall prevalence of depressive symptoms in patients with advanced schistosomiasis in China. A total of 11 studies with 1,673 participants were included. The meta results showed that the pooled prevalence of depressive symptoms in the affected patients in China was 62.01% (95% CI: 51.30% - 72.72%). The prediction interval estimated the range of the true prevalence to be 20.35% to 100%, indicating that the true prevalence in 95% of comparable populations falls within this interval. Subgroup analyses showed that high heterogeneity in the prevalence of depressive symptoms was found to be influenced by the level of depression and accompanied complications, but not in various aspects of the study methodology, including the study period, study design, or region. In patients with advanced schistosomiasis, the pooled prevalence of depressive symptoms was higher in married than in unmarried patients and in men than in women, but neither was significant. There was a significantly high level of heterogeneity among studies, but no publication bias was found and sensitivity analysis showed the results of meta-analysis were stable.

The analysis revealed a surprisingly high prevalence of depressive symptoms in advanced schistosomiasis in China. This was significantly higher than those found in other common chronic diseases, for example, in cancer patients. The research by Ding *et al*. [49] reported the pooled depression prevalence of 44.63% (95% CI: 42.24%-47.01%) in cancers in mainland China. Depression was more prevalent in advanced schistosomiasis than in cancers may be reasonable. The clinical symptoms of advanced schistosomiasis are varied and complex, including anemia, stunted growth, genital damage, irreversible organ damage, and so on [50,51], and are therefore difficult to be treated. Moreover, unacceptably high use of ineffective therapies or inappropriate changes in care may lead to some complications such as upper gastrointestinal bleeding (UGIB) [9], the most important cause of death (over 50%) in advanced schistosomiasis [18]. Anemia is a common complication and is also highly correlated with the risk of death [52]. Advanced schistosomiasis patients with anemia would still feel impaired in physical fitness and work capacity even though they had reached a status of "clinical cure" [53]. According to Jia *et al*. [21], impaired mobility (31.6%) and self-care (30.7%) were common in patients with advanced schistosomiasis. The loss of income from manual labor and the cost of necessary treatment has caused their financial stress. The patients suffer physically and/or feel stressed psychologically, finally leading to the outcome of depression. People with depression usually have higher levels of financial stress, impaired physical or work functioning and poor health [54]. In our research, we did observe that the pooled prevalence of depressive symptoms in patients was also significantly associated with complications of the disease. The high prevalence of depression problem was also reported for schistosomiasis caused by other African schistosomes. For example, in Uganda, 88% (89/103) of *S*. *mansoni* patients with UGIB and hepatic schistosomiasis experienced depression [55]. As this kind of research is limited in Africa where the highest burden of schistosomiasis exists, more work is needed.

We found that the pooled prevalence of the severe depression was 5.68%, but the mild or moderate depression was up to 55.56% from the subgroup analyses of four articles using the instrument tool SDS, which is compiled by Zung *et al*. [56]. This could be partially explained by the reason that depression could worse over time. It should be recommended that to prevent further progression to a severe level or to reduce the damage of depression, there are intervention measures that could/should be put into practice. Research on experimental animals with advanced schistosomiasis revealed that social and environmental factors, rather than the pathological process, could be the main causes of the mental problems [57]. Indeed, in China, a national aid-program to provide free medical treatment to the patients with advanced schistosomiasis has been carried out since 2004, but the subsidy could not cover the entire cost [10]. Psychological interventions could help reduce advanced schistosomiasis patients’ mental symptoms and promote their rehabilitation [41,58,59]. However, such interventions have not been effectively and widely integrated into the currently ongoing aid-program in China. Our research here emphasized a critical need that psychological interventions, together with more financial support should be given to advanced patients.

Schistosomiasis is one of about 20 diseases that are listed by the World Health Organization (WHO) as the group of NTDs [60]. NTDs are infections prevail in tropical and subtropical regions and are invariably linked to poverty, affecting at least 1 billion individuals [61,62]. The NTDs policy currently priorities disease eradication, instead of acknowledging its associated psychological consequences [63]. Those suffering from the infection were stigmatized and were even looked down upon [64,65]. It is predicted that by the year 2030, mental health conditions will be the leading burden of DALYs (disability-adjusted life years) [66]. However, research on mental health in patients caused by *Schistosoma* species (including *S*. *japonicum*, *S*. *mansoni* and *S*. *haematobium*) infections are very limited [67,68], when compared to some other NTDs like lymphatic filariasis [69]. This would warrant further research.

### Limitations

We certainly acknowledged the following limitations of our study. Firstly, although the quality of all included studies for this meta-analysis was rated as high, high heterogeneity was observed in this meta-analysis. Even after subgroup analyses, there was still a high degree of heterogeneity within each subgroup, which meant that some other associated factors were not identified. Secondly, no subgroup analysis could be conducted based on types of advanced schistosomiasis or the education level of patients as there was no data available. Future studies of this kind should take into account these potential modifying effects. Thirdly, we have exclusively considered peer-reviewed studies and not included any grey literature, potentially causing the omission of research, such as those found in government reports. Finally, all included studies were from lake and marshland areas (i.e., Hunan, Hubei, Jiangxi and Zhejiang) but not from mountainous areas (i.e., Sichuan and Yunnan), which may lead to bias in generalization.

## Conclusions

Our study highlighted the considerably high prevalence (62.01%, 95% CI: 51.30% - 72.72%) of depressive symptoms in patients with advanced schistosomiasis in China. The prevalence estimated varied significantly with depression level (mild, moderate or severe) or complications (yes or no). It is strongly recommended that more attention should be paid on the mental health of advanced schistosomiasis patients, especially those with complications. Psychological interventions, if integrated into the ongoing medical treatment program in China (and even in the world) would be very helpful in preventing or/and reducing depressive symptoms and then improving the quality of life of the targeted patients. These findings suggest that there may be unmet mental health needs in relation to schistosomiasis (and other NTDs), particularly in poor developing countries.

## Supporting information

S1 Fig Forest plot of depressive symptoms prevalence stratified by study period. (TIF)

S2 Fig Forest plot of depressive symptoms prevalence stratified by study design. (TIF)

S3 Fig Forest plot of depressive symptoms prevalence stratified by diagnostic methods. (TIF)

S4 Fig Forest plot of depressive symptoms prevalence stratified by regions of study setting. (TIF)

S5 Fig Forest plot of depressive symptoms prevalence stratified by gender of participants. (TIF)

S6 Fig Forest plot of depressive symptoms prevalence stratified by marital status of participants. (TIF)

S1 Table PRISMA checklist. (DOCX)

S2 Table Quality assessment results of all included publications. (DOCX)

S1 Text Full-text articles retrieved and excluded. (DOCX)

S2 Text Interpretation of scores for depression in the standard scales used. (DOCX)

S3 Text Search strategy. (DOCX)
