# Supplementary figures and images for: Prevalence of depressive symptoms in patients with advanced schistosomiasis in China: A systematic review and meta-analysis
Source: PLoS Negl Trop Dis. 2024 Mar 7;18(3):e0012003. doi: 10.1371/journal.pntd.0012003 (PMC10950241; doi:10.1371/journal.pntd.0012003)

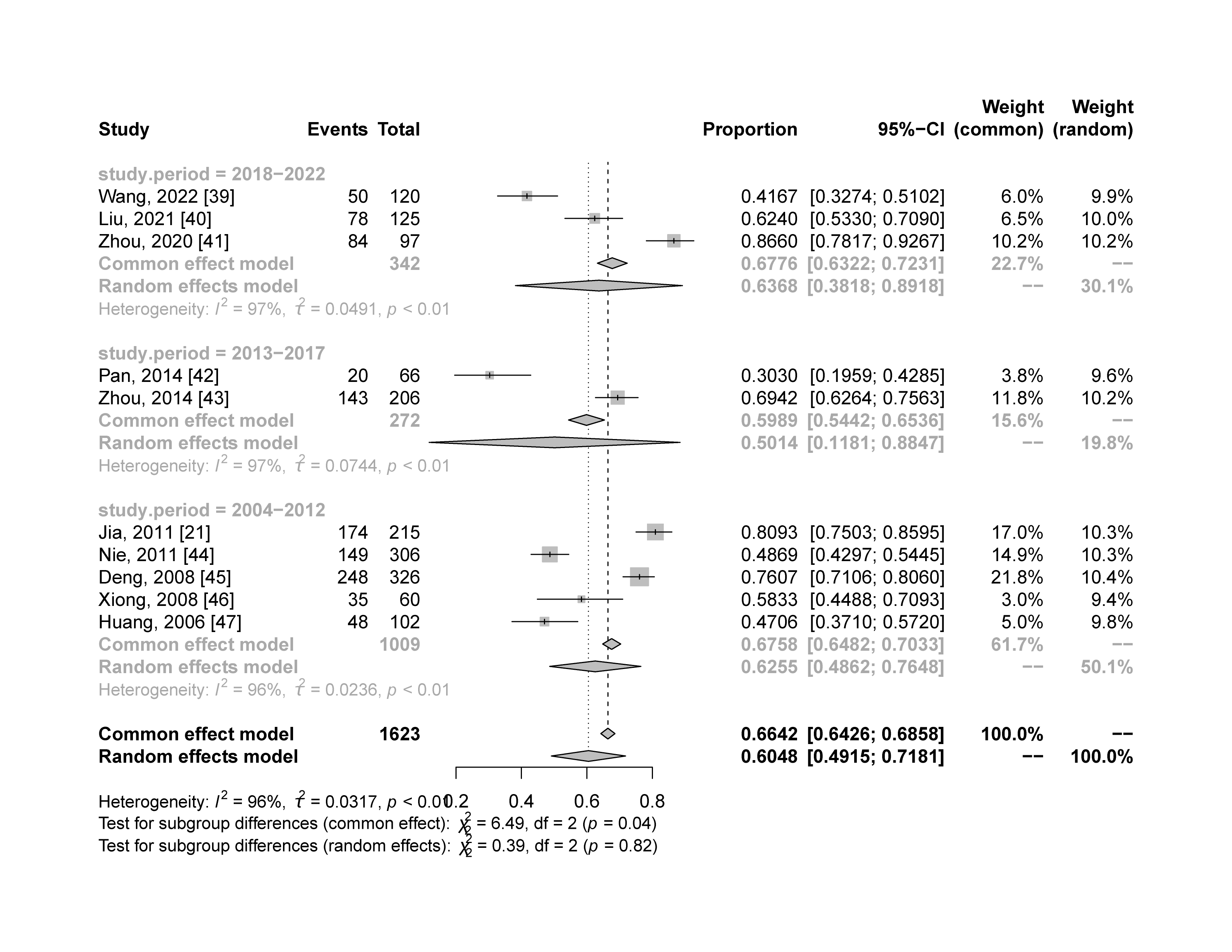

Supplement: S1 Fig — (TIF) [file pntd.0012003.s001.tif]

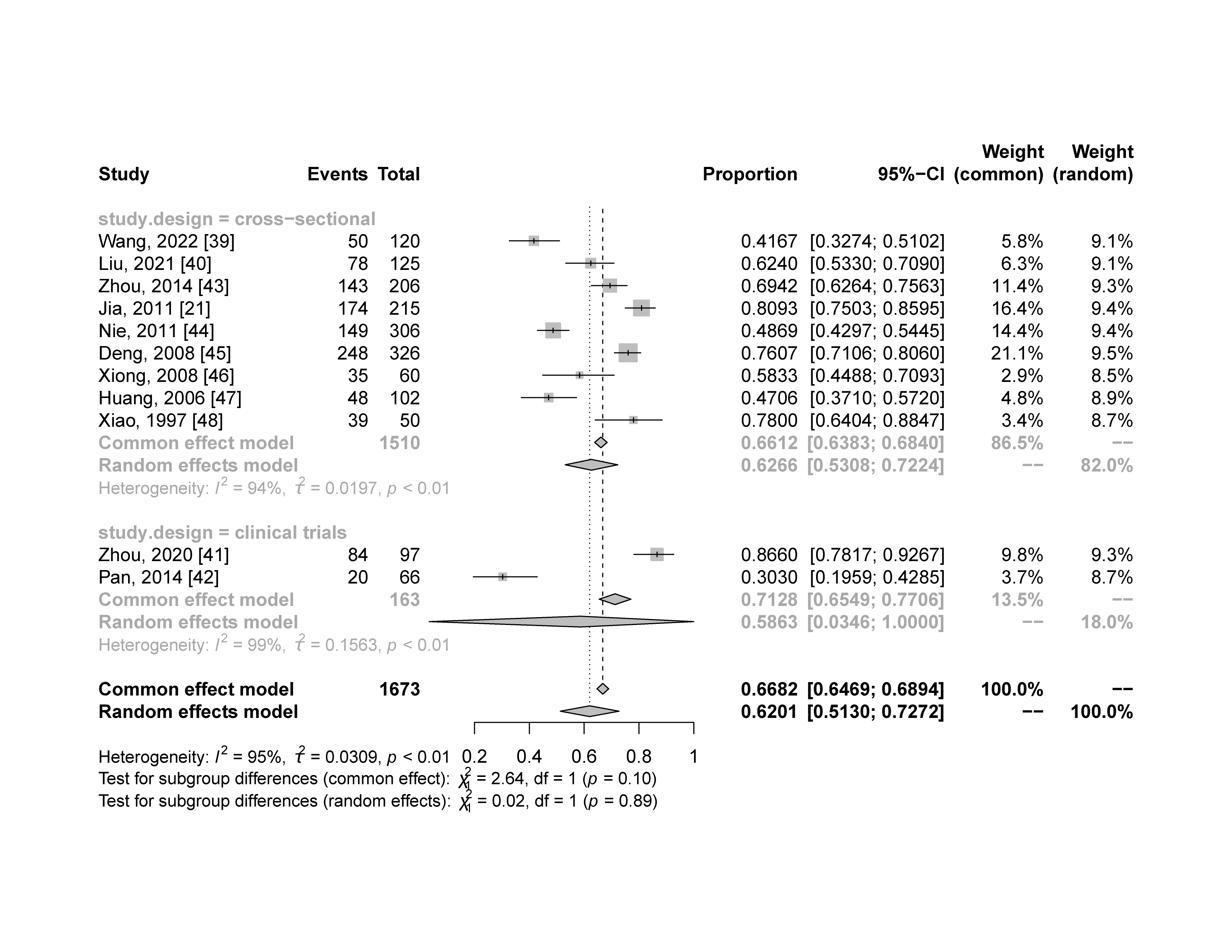

Supplement: S2 Fig — (TIF) [file pntd.0012003.s002.tif]

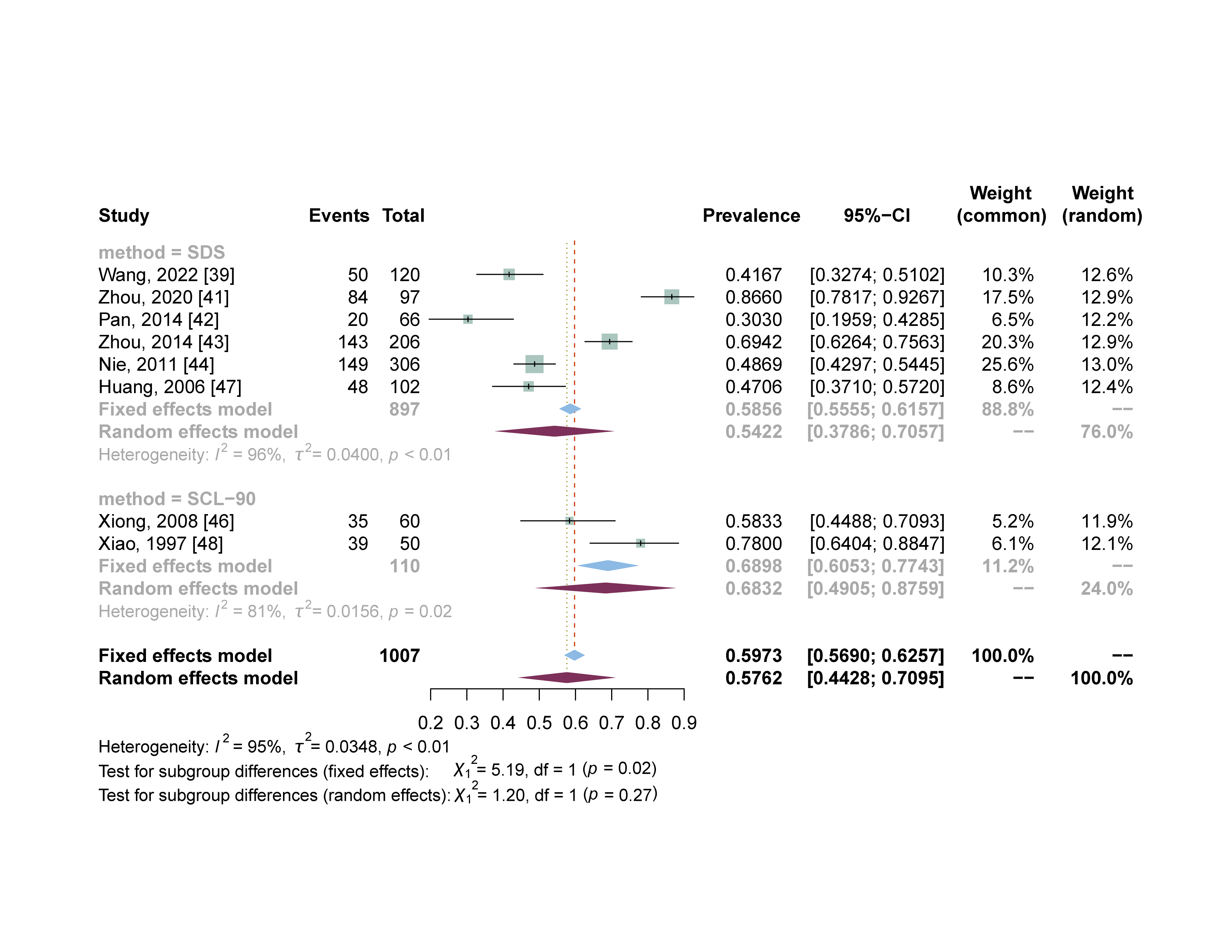

Supplement: S3 Fig — (TIF) [file pntd.0012003.s003.tif]

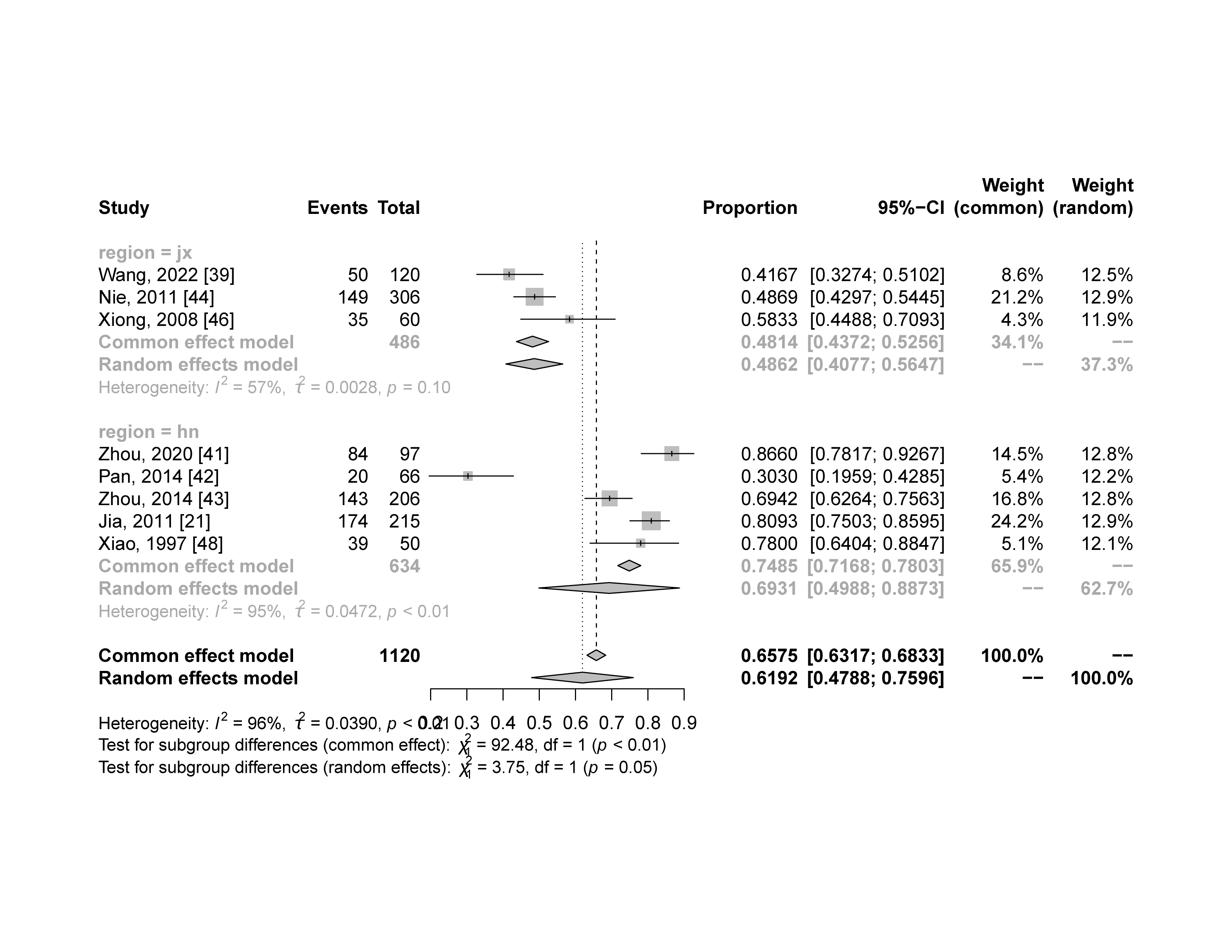

Supplement: S4 Fig — (TIF) [file pntd.0012003.s004.tif]

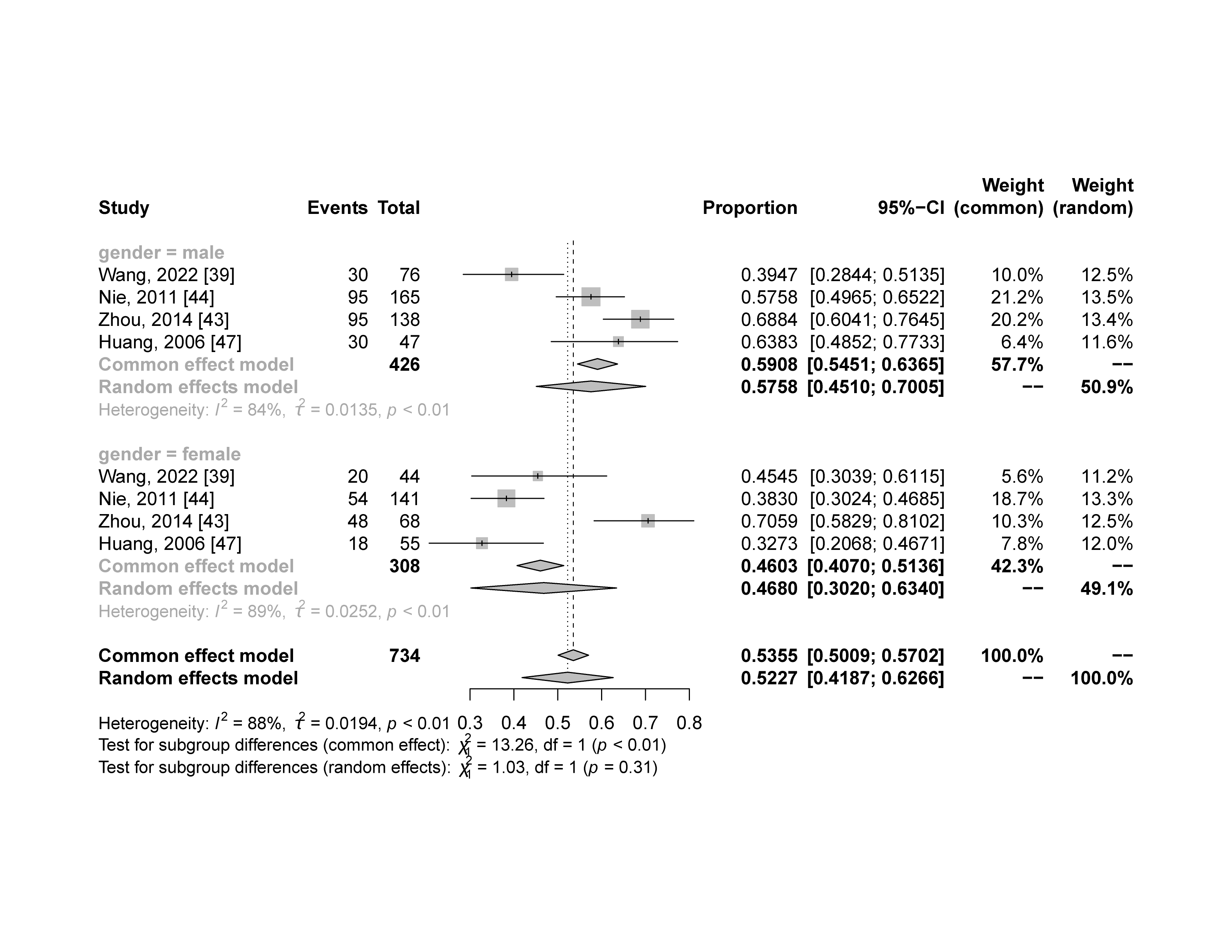

Supplement: S5 Fig — (TIF) [file pntd.0012003.s005.tif]

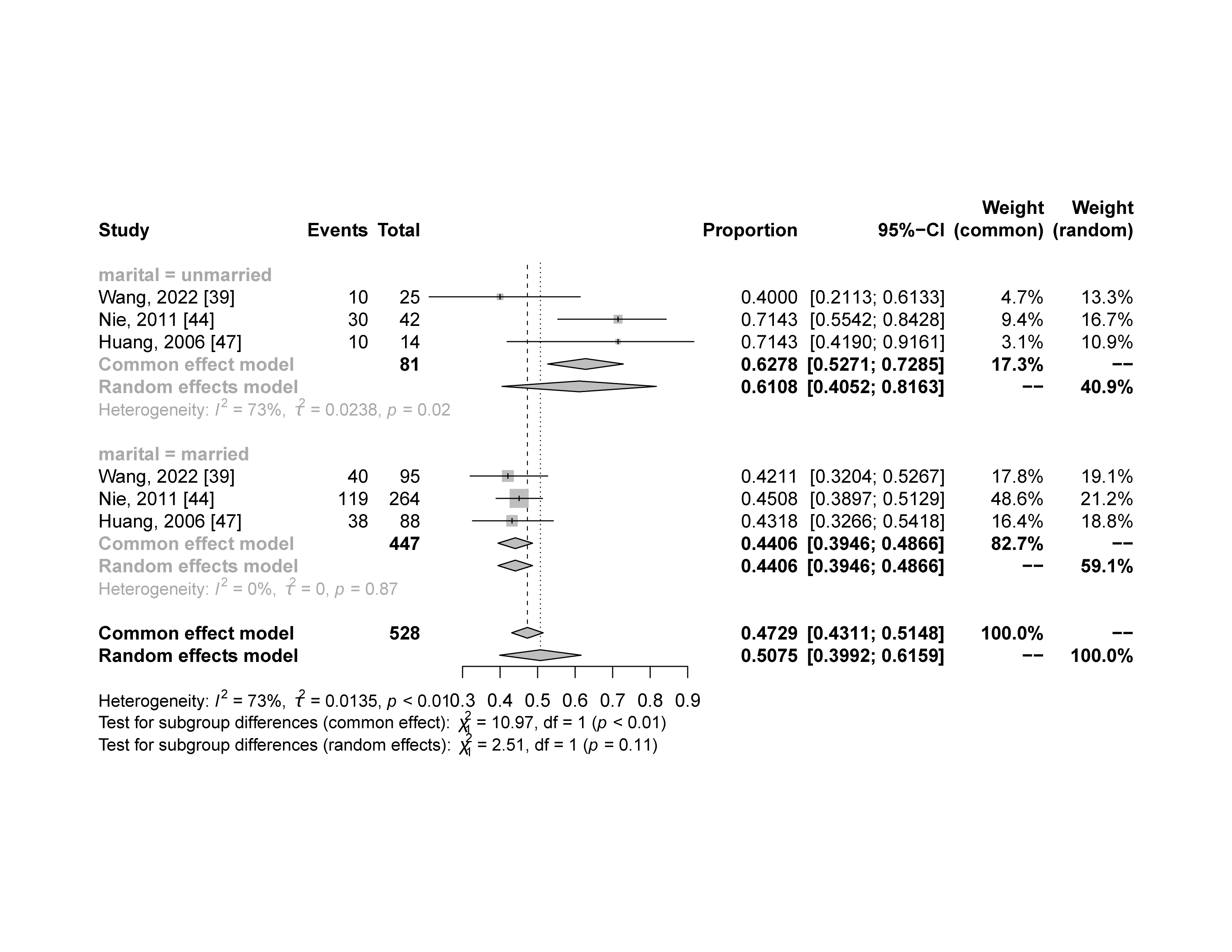

Supplement: S6 Fig — (TIF) [file pntd.0012003.s006.tif]
