# Supplementary material for: Prevalence of depressive symptoms in patients with advanced schistosomiasis in China: A systematic review and meta-analysis
Source: PLoS Negl Trop Dis. 2024 Mar 7;18(3):e0012003. doi: 10.1371/journal.pntd.0012003 (PMC10950241; doi:10.1371/journal.pntd.0012003)
Supplement: S2 Table — (DOCX) [file pntd.0012003.s008.docx]

Table S2. Quality assessment results of all included publications.

| **SN** | **Author year** | **Quality criteria score** | | | | | | | | | |
| --- | --- | --- | --- | --- | --- | --- | --- | --- | --- | --- | --- |
|  |  | **A** | **B** | **C** | **D** | **E** | **F** | **G** | **H** | **I** | **Total** |
| 1 | Wang, 2022[1] | 0 | 0 | 1 | 1 | 1 | 1 | 1 | 1 | 1 | 7 |
| 2 | Nie, 2011[2] | 0 | 0 | 1 | 1 | 1 | 1 | 1 | 1 | 1 | 7 |
| 3 | Zhou, 2020[3] | 0 | 0 | 1 | 1 | 1 | 1 | 1 | 1 | 1 | 7 |
| 4 | Zhou, 2014[4] | 0 | 0 | 1 | 1 | 1 | 1 | 1 | 1 | 1 | 7 |
| 5 | Pan, 2014[5] | 0 | 1 | 1 | 1 | 1 | 1 | 1 | 1 | 1 | 8 |
| 6 | Huang, 2006[6] | 0 | 0 | 1 | 1 | 1 | 1 | 1 | 1 | 1 | 7 |
| 7 | Xiao, 1997[7] | 0 | 0 | 1 | 1 | 1 | 1 | 1 | 1 | 1 | 7 |
| 8 | Deng, 2008[8] | 1 | 1 | 1 | 1 | 1 | 1 | 0 | 1 | 1 | 8 |
| 9 | Liu, 2021[9] | 0 | 0 | 1 | 1 | 1 | 1 | 1 | 1 | 1 | 7 |
| 10 | Jia, 2011[10] | 0 | 1 | 1 | 1 | 1 | 1 | 1 | 1 | 1 | 8 |
| 11 | Xiong, 2008[11] | 0 | 0 | 1 | 1 | 1 | 1 | 1 | 1 | 1 | 7 |
| **Average** | | | | | | | | | | | 7.2 |

Note:

1. Representativeness of the sample frame to the target population.
2. Appropriateness of the way used to sample study participants.
3. Adequateness of the sample size.
4. Description of the study subjects and settings in detail.
5. Conducted with sufficient coverage of the identified sample.
6. Validity of method used to diagnose depression.
7. The condition was measured in a standard, reliable way for all participants.
8. Appropriateness of statistical analysis.
9. Adequateness of the response rate adequate or the appropriateness of low response rate management.

**References**

1. Wang XQ. Analysis of anxiety and depression status of patients with advanced schistosomiasis and its influencing factors. Chinese General Practice Nursing. 2022;20(34):4891-4893.
2. Nie YX. Depression prevalence and quality of life of patients with advanced schistosomiasis. Chinese Journal of Schistosomiasis Control. 2011;23(5):579-581.
3. Zhou RH, Yu HQ, Liu JX, Xiao CL, Pan J, Lai RY, et al. Effect of rational emotive therapy on negative emotion in advanced schistosomiasis patients with repeated hospitalization. Chinese Journal of Schistosomiasis Control. 2020;32(03):308-310.
4. Zhou RH, Pan J, Liu KF, Shao ZW, Lai RY, Yu HQ. Quality of life and factors influencing depression in patients with advanced schistosomiasis. Journal of Nursing. 2014;21(16):65-68.
5. Pan J, Shao ZW, Liu L, Zhou RH, Yu HQ. Study on the effect of psychological intervention on negative emotions in patients with advanced schistosomiasis of the giant spleen type in the perioperative period. Modern Nurse. 2014(11):132-134.
6. Huang HY, Li M, Yang HH, Chen ML, Chen HY, Li Y. Survey on the prevalence of depression in rural patients with advanced schistosomiasis and intervention. China Health Vison. 2006;14(6):2.
7. Xiao YX, Zhang HY, Yue CY, Li YM, Chen MY, Pei GR. Analysis of the SCL-90 scale in 50 patients with advanced schistosomiasis. Chinese Journal of Schistosomiasis Control. 1997;(03):186.
8. Deng Y. The study on disease burden of advanced schistosomiasis and the quality of life of patients. MA. Thesis, Chinese center for disease control and prevention. 2008. Available from: https://kns.cnki.net/KCMS/detail/detail.aspx?dbname=CMFD201902&filename=1019196036.nh
9. Liu R, Zhang JF, Yan XL, Wen LY. Comparative study of SF-36 and EQ-5D-5L in evaluating quality of life for patients with advanced schistosomiasis. Chinese Journal of Parasitology and Parasitic Diseases. 2021;39(05):639-646.
10. Jia TW, Utzinger J, Deng Y, Yang K, Li YY, Zhu JH, et al. Quantifying quality of life and disability of patients with advanced *schistosomiasis japonica*. PLoS Negl Trop Dis. 2011;5(2):e966.
11. Xiong YK, Xiong YM, Wan LH. Study of the psychological status and living quality in patients with advanced schistosomiasis. Chinese Journal of Schistosomiasis Control. 2008;(04):324+262.
