# Supplementary material for: Prevalence of depressive symptoms in patients with advanced schistosomiasis in China: A systematic review and meta-analysis
Source: PLoS Negl Trop Dis. 2024 Mar 7;18(3):e0012003. doi: 10.1371/journal.pntd.0012003 (PMC10950241; doi:10.1371/journal.pntd.0012003)
Supplement: S1 Text — (DOCX) [file pntd.0012003.s009.docx]

The list of the 37 full-text articles excluded

1. Without exact data or insufficient data 25

2. Not diagnosed by standard method 10

3. Duplicate data 2

[1] Zhang GZ, Zhang Y, Huang NL, et al. Application effect of continuous nursing on advanced schistosomiasis. Journal of Tropical Diseases and Parasitology. 2020;18(02):108-110.

**Reason: Depressive symptoms were not diagnosed through standard questionnaires.**

[2] Xu HY. Investigation on quality of life of patients with advanced schistosomiasis in Chaisang District of Jiujiang City and analysis of its influencing factors. China Modern Medicine. 2022;29(18):128-132.

**Reason: Without exact data on the prevalence of depressive symptoms.**

[3] Wang JP, Wu YM, Wang XJ. Analysis of self-perceived burden in patients with advanced schistosomiasis. Chinese Rural Health Service Administration. 2017;37(02):194-196.

**Reason: Depressive symptoms were not diagnosed through standard questionnaires, but rather through personal visits and discussions.**

[4] Guo JD, Li QY, Yin XM, et al. Analysis of the quality-of-life scale of patients with advanced schistosomiasis. Chinese Journal of Behavioral Medicine and Brain Science. 2002;(04):23-25.

**Reason: Depressive symptoms were not diagnosed through standard questionnaires.**

[5] Zhang BR, Xiao MQ, Yan JQ, et al. Investigation of mental states of bilharziasis patents in new epidemic area. Journal of Clinical Psychosomatic Diseases. 2008;(01):47-48.

**Reason: Without exact data on the prevalence of depressive symptoms of advanced schistosomiasis patients.**

[6] Zhang BR, Hu B, Li W, et al. Analysis of 300 cases of schistosomiasis patients in newly infected areas with local residents and Chinese normative SCL-90 ratings. Medical Journal of Chinese People's Health. 2009;21(24):3119,3157.

**Reason: Without exact data on the prevalence of depressive symptoms of advanced schistosomiasis patients.**

[7] Zhou RH, Lai RY, Xiao SY, et al. Study on the effect of psychological intervention on patients with advanced schistosomiasis and negative emotion. Practical Preventive Medicine. 2014;21(12):1426-1428+1415.

**Reason: Without exact data on the prevalence of depressive symptoms.**

[8] Yang L, Liu JJ. Psychological interventions to improve negative emotions in perioperative patients with advanced schistosomiasis. Chinese Journal of Modern Nursing. 2013,19(13):1526-1527.

**Reason: Without exact data on the prevalence of depressive symptoms.**

[9] Xu HY. Effectiveness of combined nursing interventions on patients with advanced schistosomiasis combined with depression. World Latest Medicine Information. 2021,21(69):343-344,346.

**Reason: Without exact data on the prevalence of depressive symptoms.**

[10] Wu EM. Analysis of the impact of nursing interventions on the improvement of quality of life of patients with advanced schistosomiasis. For all Health. 2014,8(12):277-278.

**Reason: Depressive symptoms were not diagnosed through standard questionnaires.**

[11] Qin ZF. Psychological analysis and countermeasures of patients with advanced schistosomiasis. Guide of China Medicine. 2010,8(35):313-314.

**Reason: Depressive symptoms were not diagnosed through standard questionnaires.**

[12] Shi HX. Psychological characteristics of schistosomiasis patients and nursing countermeasures. World Latest Medicine Information. 2019,19(55):285+287.

**Reason: Without exact data on the prevalence of depressive symptoms.**

[13] Ding X, Wang F, Rong XB, et al. Effect evaluation of quality of life of advanced schistosomiasis patients. Chinese Journal of Social Medicine. 2010;27(06):384-385+388.

**Reason: Without exact data on the prevalence of depressive symptoms.**

[14] Nie YX. Efficacy of the combination of tandospirone and escitalopram in the treatment of patients with advanced schistosomiasis with depression. Jiangxi Medical Journal. 2019,54(07):835-837.

**Reason: Without exact data on the prevalence of depressive symptoms.**

[15] Luo XL. A comprehensive intervention study on the quality of survival of patients with advanced schistosomiasis in Xiaofeng region. Chinese Journal of Public Health Management. 2012,28(01):83-84.

**Reason: Without exact data on the prevalence of depressive symptoms.**

[16] Hua HY, You H, Zhang Y. Evaluation of quality of life in advanced schistosomiasis patients in Jiangsu Province. Chinese Journal of Schistosomiasis Control. 2010,22(06):562-566.

**Reason: Depressive symptoms were not diagnosed through standard questionnaires.**

[17] Li LZ. Comfort care in the perioperative period of patients with advanced schistosomiasis of the megasplenic type. Medical Aesthetics and Cosmetology. 2015,(2):485-485,486.

**Reason: Without exact data on the prevalence of depressive symptoms.**

[18] Lai RY, Shao ZW, Yu HQ, et al. Design and application of psychological intervention paths for ascites type of advanced schistosomiasis patients. Chinese Journal of Schistosomiasis Control. 2014,26(06):662-664.

**Reason: Without exact data on the prevalence of depressive symptoms.**

[19] Lai RY. Observation on the effect of perioperative nursing intervention in advanced schistosomiasis patients with megalosplenia. Journal of Tropical Diseases and Parasitology. 2021,19(01):39-40+46.

**Reason: Without exact data on the prevalence of depressive symptoms.**

[20] Zhang XY. The study on the economic burden and impacts of advanced schistosomiasis in Yunnan Province. MA. Thesis, Dali University. 2011. Available from: https://kns.cnki.net/kcms2/article/abstract?v=j6HAoO1nZAx_Ynk7nvV8AVdeIoaIcLB68SwINBuPCco80vjuviJjXu45XqdLehkm1Z4CNZE0i1LPOsWKkgffjCPRf6jR_QzET-QMO08l3JpFxOMveyyybq1_zQ0hDj_0IIpwVAtruHZMvIuSXwlxzg==uniplatform=NZKPTlanguage=CHS

**Reason: Without exact data on the prevalence of depressive symptoms.**

[21] Fang XP. A survey of the psychological state of schistosomiasis patients and an analysis of the factors influencing it. Modern Nurse. 2005,(08):69-70.

**Reason: Without exact data on the prevalence of depressive symptoms of advanced but chronic schistosomiasis patients.**

[22] Zuo QL. Evaluation of the effect of comprehensive nursing intervention on improving the quality of life of patients with advanced schistosomiasis. Medical Journal of Chinese People’s Health. 2014,26(24):126-127.

**Reason: Without exact data on the prevalence of depressive symptoms.**

[23] Zhu L. Analysis of the effect of nursing interventions to improve the quality of life of patients with advanced schistosomiasis. World Latest Medicine Information. 2016,16(57):270.

**Reason: Without exact data on the prevalence of depressive symptoms.**

[24] Chen ML, Chen YY, Li Y, et al. Nursing care of 79 patients with advanced schistosomiasis. Journal of Tropical Diseases and Parasitology. 2016,14(02):104-105.

**Reason: Depressive symptoms were not diagnosed through standard questionnaires.**

[25] Ding X, Wang F, Rong XB, et al. Effect evaluation of assistance on advanced schistosomiasis based on improvement of quality of life and satisfaction of advanced schistosomiasis patients. Chinese Primary Health Care. 2011,25(03):68-71.

**Reason: Without exact data on the prevalence of depressive symptoms.**

[26] Xiao Y, Dai YH, Zhu HG, et al. Effect of medical treatment for advanced schistosomiasis in Hubei Province, 2004-2007. Chinese Journal of Schistosomiasis Control. 2008,20(06):467-468.

**Reason: Without exact data on the prevalence of depressive symptoms.**

[27] Zhou RH, Xiao CL, Chen GM et al. Survey and analysis on the health self-management skills in patients with advanced schistosomiasis in Hunan province. Journal of Tropical Diseases and Parasitology. 2018,16(04):213-216.

**Reason: Without exact data on the prevalence of depressive symptoms.**

[28] Zhang LZ. Psychological characteristics and nursing countermeasures of schistosomiasis patients. Women's Health Research. 2015;(18):105,96.

**Reason: Without exact data on the prevalence of depressive symptoms of advanced schistosomiasis patients.**

[29] Li M, Fu CL. Psychological characteristics and nursing countermeasures of patients with advanced schistosomiasis. World Latest Medicine Information. 2016;16(65):295-296.

**Reason: Without exact data on the prevalence of depressive symptoms.**

[30] Jiang HF, Meng SF. Application of focused solution mode in the treatment of advanced schistosomiasis. China Continuing Medical Education. 2019;11(33):163-165.

**Reason: Without exact data on the prevalence of depressive symptoms.**

[31] Fan XC, Jiang LY, Liu WX. Nursing of advanced schistosomiasis patients: a report of 52 cases. Chinese Journal of Schistosomiasis Control. 2014;26(03):345+348.

**Reason: Depressive symptoms were not diagnosed through standard questionnaires.**

[32] Li YY, Zhou YB. A comparative study of two hospitalizations in patients with advanced schistosomiasis of the ascites type. Chinese Journal of Schistosomiasis Control. 2002;(01):55-56.

**Reason: Without exact data on the prevalence of depressive symptoms.**

[33] You H, Hua HY, Chen JF, et al. Application of SF-36 scale among patients with advanced schistosomiasis in Jiangsu province. Chinese Journal of Epidemiology. 2012,33(8):803-807.

**Reason: Depressive symptoms were not diagnosed through standard questionnaires.**

[34] Zhou RH, Pan J, Xiao SY, et al. Analysis on depression of patients with advanced schistosomiasis and its influencing factors. Chinese Journal of Schistosomiasis Control. 2014,26(03):270-271.

**Reason: Duplicate data was found in two papers of the same researcher (Journal of Nursing, 2014, 21(16):65-68; Chinese Journal of Schistosomiasis Control, 2014, 26(03):270-271) and finally we chose the paper with the most complete data of depression level (Journal of Nursing, 2014, 21(16):65-68).**

[35] Pan J, Zhou RH, Liu KF, et al. Investigation on anxiety, depression and life quality of hospitalized patients with advanced schistosomiasis and related influencing factors. Journal of Tropical Medicine. 2014,14(08):1082-1085+1092.

**Reason: Duplicate data was found in the paper of Pan (Journal of Tropical Medicine, 2014, 14(08):1082-1085+1092) and Zhou (Journal of Nursing, 2014, 21(16):65-68), so we chose more recent (Journal of Nursing, 2014, 21(16):65-68) in these two studies.**

[36] Nie YX, Xu TL. Cognitive behavioral therapy for depression in advanced schistosomiasis patients. Chinese Journal of Schistosomiasis Control. 2012,24(04):471-473.

**Reason: Without exact data on the prevalence of depressive symptoms.**

[37]Gao SH, Hu CM. Psychological care of patients with portal hypertension in advanced schistosomiasis. Parasitoses and Infectious Diseases. 1999,(S1):21.

**Reason: Depressive symptoms were not diagnosed through standard questionnaires.**
