# Supplementary material for: Prevalence of depressive symptoms in patients with advanced schistosomiasis in China: A systematic review and meta-analysis
Source: PLoS Negl Trop Dis. 2024 Mar 7;18(3):e0012003. doi: 10.1371/journal.pntd.0012003 (PMC10950241; doi:10.1371/journal.pntd.0012003)
Supplement: S2 Text — (DOCX) [file pntd.0012003.s010.docx]

The interpretation of scores for depression in the standard scales used

Table 1 The interpretation of SDS scores [1].

|  | Within normal range | Minimal to mild depression | Moderate to severe depression | Severe depression |
| --- | --- | --- | --- | --- |
| SDS | < 50 | 50 – 59 | 60 – 69 | > 70 |

Table 2 The interpretation of SCL-90 scores.

|  | No problem | Moderate problem | Psychological problem |
| --- | --- | --- | --- |
| SCL-90 | < 0.5 | 0.5 – 1 | > 1 |

* When the total score determined for each subtest (somatization, obsessive-compulsive, interpersonal sensitivity, **depression**, anxiety, anger-hostility, phobic anxiety, paranoid thought and psychosis) is divided by the number of items in that subtest, a score is obtained for that dimension. The General Symptom Index (GSI) identifies symptom distribution by dividing the scores of all items by the total number of questions [2].

Table 3 The ten most commonly reported EQ-5D health states [www.euroqol.org].

| EQ-5D | Description |
| --- | --- |
| 11111 | Full health/no problems reported |
| 11121 | Moderate problems: pain/discomfort |
| 21121 | Moderate problems: mobility pain/discomfort |
| 11112 | Moderate problems: anxiety/depression |
| 11122 | Moderate problems: pain/discomfort anxiety/depression |
| 21122 | Moderate problems: mobility pain/discomfort anxiety/depression |
| 11222 | Moderate problems: usual activities pain/discomfort anxiety/depression |
| 21222 | Moderate problems: mobility usual activities pain/discomfort anxiety/depression |
| 11221 | Moderate problems: usual activities pain/discomfort |
| 11123 | Moderate problems: pain/discomfort Severe problems: anxiety/depression |

* EQ-5D is based on a descriptive system that defines health in terms of 5 dimensions: Mobility, Self-Care, Usual Activities, Pain/Discomfort, and Anxiety/**Depression** [3]. Each dimension has 3 response categories corresponding to no problems, some problems, and extreme problems. This results in a five-dimensional, three-level health measurement system that can be used as a five-digit set of numbers to provide a comprehensive picture of the respondent’s health status on the day in question [4].

**Reference**

[1] Sepehry, A.A. Self-Rating Depression Scale (SDS). Encyclopedia of Quality of Life and Well-Being Research. https://doi.org/10.1007/978-94-007-0753-5_2641

[2] Mandana H, Ozgur K, Ibrahim I, et al. Should we screen patients’ relatives for psychological status, signs and symptoms in the emergency department? A cross-sectional survey using the Symptom Check List (SCL-90). Signa Vitae. 2021;17(2):139-144. doi: 10.22514/sv.2021.004

[3] Brooks R. EuroQol: the current state of play. Health Policy. 1996;37(1):53-72. doi: 10.1016/0168-8510(96)00822-6.

[4] Nordlund A, Ekberg K, Kristenson M. EQ-5D in a general population survey--a description of the most commonly reported EQ-5D health states using the SF-36. Qual Life Res. 2005;14(4):1099-109. doi: 10.1007/s11136-004-3062-2.
