# Supplementary material for: Prevalence of depressive symptoms in patients with advanced schistosomiasis in China: A systematic review and meta-analysis
Source: PLoS Negl Trop Dis. 2024 Mar 7;18(3):e0012003. doi: 10.1371/journal.pntd.0012003 (PMC10950241; doi:10.1371/journal.pntd.0012003)
Supplement: S3 Text — (DOCX) [file pntd.0012003.s011.docx]

**Search strategy**

**Full search strategy for each database**

CNKI (in Chinese)

(Topic: wanqixuexichongbing) AND (Title/Abstract/Keywords: yiyu) OR (Title/Abstract/Keywords: xinli)

WanFang Data (in Chinese)

Topic:(wanqixuexichongbing) and Topic:(yiyu)

CQVIP

M=wanqixuexichongbing AND （K=yiyu OR xinli）

PubMed

(((depression[Title/Abstract]) OR (depressive symptom[Title/Abstract])) OR (depressive disorder[MeSH Terms])) AND (advanced schistosomiasis[Title/Abstract])

Web of Science

((TS=(depression)) OR TS=(depressive symptom)) AND TS=(advanced schistosomiasis)

The Cochrane Library

(("depressive disorder"):ti,ab,kw OR ("depression")):ti,ab,kw AND ("advanced schistosomiasis"):ti,ab,kw
